# Perceptions, motivations, and beliefs about HIV risk and pre-exposure prophylaxis (PrEP) among participants in a nurse-led PrEP service (PrEP-RN)

**DOI:** 10.1186/s12879-022-07146-3

**Published:** 2022-02-28

**Authors:** Lauren Orser, Patrick O’Byrne, Dave Holmes

**Affiliations:** 1 Faculty of Health Sciences, School of Nursing, University of Ottawa, Ottawa, ON Canada; 2 Sexual Health and Harm Reduction Services, Ottawa Public Health, Ottawa, ON Canada

**Keywords:** HIV, Pre-exposure prophylaxis, HIV prevention, Nursing

## Abstract

**Background:**

While HIV pre-exposure prophylaxis (PrEP) has become more readily available in Canada, its uptake among HIV priority populations continues to be affected by system-level and individual factors. Such impediments relate to challenges by healthcare providers in assessing HIV-related risk and variability in patients’ motivations for PrEP initiation and continued engagement in care.

**Methods:**

In Ottawa, Canada, a group of researchers implemented Canada’s first nurse-led HIV prevention program, known as PrEP-RN. As part of this pilot, qualitative interviews were completed with fourteen patients who had accessed PrEP-RN. The purpose of these interviews was to understand participants’ perspectives related to HIV prevention and experiences accessing care through a nurse-led service. Interviews were analyzed using thematic analysis, which were organized into the two major themes of (1) motivations for PrEP initiation and (2) beliefs about the benefits of PrEP.

**Results:**

Findings revealed participants’ motivations for PrEP differed from healthcare provider’s views of risk, which were influenced by external life factors and personal perceptions of risk. In addition, participants discussed the benefits of PrEP in terms of its ability to manage their potential mistrust of sexual partners, control their sexual health, and liberate fears and anxieties related to HIV.

**Conclusions:**

Based on these findings, health and allied providers should consider incorporating individual motivations and beliefs into patient education and counselling about PrEP to better target HIV prevention care at persons are at elevated risk of HIV. These perspectives could also be used to re-structure web and social media campaigns to increase PrEP uptake among HIV priority populations.

## Background

In Canada, over the last 10 years, a relatively stable number of HIV diagnoses continue to occur among so-called ‘priority groups’ [1], which include gay, bisexual, and other men who have sex with men (gbMSM), persons from HIV endemic regions, members of Indigenous communities, trans persons, and people who use drugs [1, 2]. Despite attempts to establish behavioural interventions to curb the rate of HIV diagnoses among the foregoing groups [3], the success of these efforts has been limited. Evaluations of risk reduction models based on condom use tend to yield poor uptake due to inaccessibility of, and personal preferences against, condoms—leading to decreased success of this approach for HIV prevention [4, 5].

Considering this, HIV pre-exposure prophylaxis (PrEP) was established as a once daily chemoprophylaxis to reduce the likelihood of HIV acquisition among persons at elevated risk of infection [6]. PrEP is antiretroviral medication that can be used for HIV treatment or for HIV prevention among persons who are HIV-negative [5]. When taken daily as prescribed, PrEP has been shown to reduce the likelihood of HIV seroconversion by up to—and possible in excess of—99% [6]. Despite the known efficacy of PrEP, the uptake and retention rates among persons at elevated risk of HIV infection has been mixed [7], resulting in minimal-to-no decreases in HIV rates throughout most Canadian provinces and territories [1, 2]. This lack of change in HIV diagnosis rates signals that more needs to be done to understand patients’ rationales for PrEP initiation and engagement in care to better target HIV prevention efforts.

From a healthcare provider perspective, it can be challenging to ascertain HIV-related risks in individual patients. On this point, current guidelines recommend PrEP be offered to persons who belong to the aforementioned priority populations [8]; however, membership in said groups does not automatically denote risk. As a result, patients with reduced risk of HIV acquisition based on HIV correlates (e.g., a gay man who reports consistent condom use during sex) might be prioritized for PrEP because they belong to a group with elevated HIV prevalence [1]. Conversely, persons within the same group who have objective risk indicators (e.g., a gay man with a sexual partner recently diagnosed with HIV) or who might be at elevated risk for HIV, but belong to a group with lower HIV prevalence (e.g., a heterosexual woman diagnosed with infectious syphilis) might be less considered for PrEP [9–11]. These nuances between individual and population-level risk indicators can make it difficult for healthcare providers to determine who might benefit from PrEP.

From a patient perspective, literature has shown that persons from priority groups can experience system-level barriers to sexual health and HIV prevention services, making it difficult for patients to (1) self-select for PrEP care and (2) engage in follow-up with healthcare providers [12, 13]. Such concerns relate to discomfort discussing sensitive health information and fears providers might engage in stigmatizing or judgemental behaviour toward them (e.g., related to sexual orientation, gender identity, sexual practices, etc.) [12–14]. In addition, ethnic minorities, such as African, Caribbean, Black and Indigenous persons face higher incidences of racial discrimination and systemic racism in healthcare settings [14–16], which can contribute to greater health inequities for these patients [13, 16].

An additional challenge to PrEP uptake among HIV priority groups is that many patients do not consider themselves to be sufficiently at-risk for HIV, and therefore, do feel that PrEP use is warranted for them [17–19]. Indeed, a qualitative study exploring patients’ rationale for declining PrEP referrals [20] found the most common reason against PrEP initiation was that many patients considered their practices to be low risk for HIV acquisition and, as a result, felt that PrEP should be for reserved for “high-risk others” ^p.1286^ (i.e., other people who engaged in condomless sex with multiple partners) [20]. Notably, among participants in this study who did accept PrEP, most identified as male and gbMSM [20]. Currently, PrEP uptake among women, trans persons, people who use drugs, and ethnic minority groups remains low, which is in part likely due to perceptions of HIV risk and inaccessibility of PrEP [17–19, 21].

These impediments to PrEP initiation and care among persons at elevated risk for HIV signals that more needs to be done to understand patients’ motivations for PrEP initiation and sustained use, so healthcare providers can more effectively target HIV prevention services. In an effort to address this gap in healthcare provider knowledge, we launched Canada’s first nurse-led PrEP service, known as PrEP-RN [22]. As part of this pilot project, we completed qualitative interviews with 14 patients who attended the PrEP-RN clinic for care. With our objective to better understand patients’ motivations for using, and personal beliefs regarding, PrEP, during our interviews we sought to understand participants’ (1) awareness of HIV prevention services, (2) rationale for PrEP initiation, (3) experience with PrEP-RN services, and (4) perspectives on HIV prevention, including factors associated with continued PrEP use.

## Methods

### Sampling: PrEP-RN

PrEP-RN was an open cohort prospective observational study that occurred in Ottawa, Canada, in a downtown sexual health clinic, run by the local public health department (Ottawa Public Health). Qualifying patients for PrEP-RN [22] were those who met objective indicators of risk identified during provincially mandated sexually transmitted infection (STI) follow-up [23] or STI screening or treatment visits at the sexual health clinic. Risk indicators were established based on known HIV correlates and included the following: a diagnosis of infectious syphilis, lymphogranuloma venereum, or two or more rectal chlamydia or gonorrhea infections, single use of HIV post-exposure prophylaxis (PEP), sexual contact with a partner recently diagnosed with HIV, or persons whom nurses felt were at elevated risk for HIV based on clinical judgement (e.g., chlamydia or gonorrhea in other anatomical sites, engagement in sex work, recent or historical diagnosis of hepatitis C). Patients who met the aforementioned criteria were offered a PrEP referral to either the PrEP-RN clinic or an alternate infectious diseases clinic in Ottawa.

As part of this pilot project, funding was obtained from the Ontario HIV Treatment Network to offer PrEP medications at a subsidized rate to uninsured patients enrolled in PrEP-RN (e.g., without government assistance or third-party insurance). Study funded medications were available for the first 3 months of PrEP, which reduced the monthly cost of the medication from $250 to $50. In addition, nurses provided information on how to register for the provincial publicly funded drug program so patients had continued coverage after the 3-month period.

### Data collection

Patients who accepted a referral to PrEP-RN were asked to sign a research consent form for collection of clinical information regarding PrEP care and to be contacted for an interview about their experiences accessing PrEP in a nurse-led service. Clinical information included, demographic information, reason for referral, HIV and serum creatine monitoring, sexually transmitted infection diagnosis during PrEP care, mental health assessment, and length of duration in care. (See O’Byrne et al., 2019 and 2020 for more information) [22, 24]. Interview recruitment occurred from August 2018 to March 2020 among participants who attended PrEP-RN services. Of the 88 active patients, 35 provided consent to be contacted by a research assistant for an interview; 14 agreed to participate. Research ethics was obtained from the University of Ottawa (H-04-18-533) with approval from Ottawa Public Health.

Interviews were completed from May 2020 to July 2020 amidst the COVID-19 pandemic. The interview format was semi-structured, where the research assistant was given a list of prompts to facilitate discussions specific to the research aims, while at the same time, allowing participants flexibility to raise points of interest or importance to them regarding their experiences using PrEP [25]. Due to COVID-19 restrictions barring in-person contact, a research ethics board approval amendment was obtained to complete all interviews via Microsoft Teams. Prior to conducting the interviews, verbal consent was obtained from participants. Interviews were video recorded during the Teams call, transcribed verbatim, anonymized by a code number, and analyzed thematically. As part of the analysis [26], we independently reviewed transcripts multiple times and portioned key segments according to the research objectives. These segments were labelled based on inferred meaning, which were documented in a codebook to capture recurrent and related labels. Similar data labels were subsequently clustered into codes, which were reviewed against the raw data to ensure the researcher’s interpretations aligned with participants’ statements. Data saturation was achieved by identifying a small number of cases that deviated from this narrative. As a team, we reviewed final codes within the context of the data to establish homogeneity and clustered these codes into major themes to produce an over-arching narrative about participants motivations for initiating PrEP and personal beliefs regarding the benefits of this intervention.

## Findings

A total of 14 participants completed qualitative interviews, including current and former PrEP-RN patients. All patients identified as male, and the majority were gay (n = 13/14). The median age of participants was 33 years (range 22–43 years). For ethnicity, 71% (n = 10/14) identified as white. In terms of reason for PrEP referral, 43% (n = 6) were due to PEP and 29% (n = 4) were based on nurses’ clinical judgement. The remaining 29% (n = 4) had the following risk factors: 1 was an HIV contact, 1 had syphilis, 1 had two rectal chlamydia infections, and 1 had multiple bacterial STI diagnoses. Of these 14 patients, 50% (n = 7) were currently using PrEP (and had been for more than 12 months), 21% (n = 3) had stopped PrEP within 6 months of initiation, and 29% (n = 4) had stopped PrEP within 2 months of initiation. (Table 1).

**Table 1:** Demographic characteristics of participants

Demographic indicator	N	%
Gender—Male	14	100
Sexual orientation—Gay	13	92.8
Ethnicity—White	10	71.4
*Reason for referral*		
PEP Use	6	42.8
Clinical Judgement	4	28.6
Other—HIV contact, Syphilis, rectal Chlamydia or Gonorrhea, multiple STIs	4	28.6

**Fig. 1:**
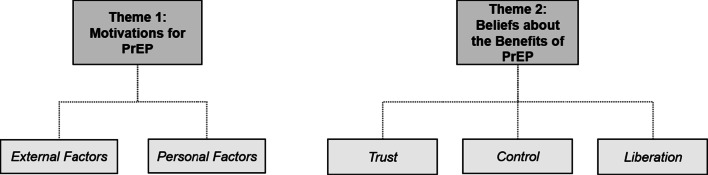
Themes and subthemes

Thematic analysis of these interviews yielded two major themes with coinciding subthemes: (1) Motivations for PrEP and (2) Beliefs about the Benefits of PrEP. (Fig. 1).

### Theme 1: motivations for PrEP

This theme explores the personal factors that motivated participants’ decisions to initiate PrEP. Findings for this section emerged from questions regarding participants’ rationale for PrEP use—specifically, those related to their STI history or sexual practices that led them to be referred for, and choose to initiate, PrEP. Notably, participants’ acknowledgement of their potential risk behaviours and subsequent reason for being referred for PrEP seemed to differ from the factors that motivated their decision to use PrEP. Motivations were, therefore, separated into the sub-themes of external factors for PrEP referral and initiation (i.e., based on the timing of PrEP offers) and personal factors for PrEP referral and initiation (i.e., based on individual perceptions of risk).

#### External factors

For many participants, the determining factor surrounding PrEP initiation related to the timing of being offered PrEP by public health nurses. In particular, participants noted being in a sufficiently good position to consider this intervention when PrEP was raised in conversation by nurses. For these individuals, readiness for PrEP related to external factors, such as access to insurance to pay for PrEP (despite the offer of study-funded medication for those without coverage) or the increasing popularity of PrEP within their social and sexual networks, which changed their views on PrEP initiation. On this, participants stated:

*My partner and I played with another couple. We started inquiring, and thought we have health insurance that covers it, and if we’re going to be doing any more of that, why would we subject ourselves to any level of risk? (P10)*

*[PrEP] is very common in the gay community, that’s why I was considering using it. (P13)*

*The hook-up apps started to show if people were on PrEP and at that point, I realized it was pretty common. I also got [access to] health insurance, so it was kind-of the perfect timing to go on PrEP. (P6)*

*I realized I could get [PrEP] 100% paid for, so I [thought] ‘it’s better to be safe than sorry’. Especially since the cost objection was removed. (P14)*

Notable in these statements, is that the motivations for PrEP initiation appeared to be context specific based on extraneous life factors. For some participants, the increasing popularity of PrEP among their peers or sexual partners led them to “consider using it”, while for others, assessments weighing risk behaviours against the cost of PrEP became the determining factor for initiation.

For other participants, the impetus to start PrEP came from discussions with healthcare providers during STI follow-up or routine screening visits. While these individuals might have been considering PrEP, it was not something they were actively seeking until nurses raised it during discussions about their sexual health. This led some participants who were previously ambivalent to consider “*get[ting] on board with PrEP”* (P6). Other participants raised similar sentiments about how a brief discussion about PrEP with nurses spurred their motivations for its initiation:

*My partner wasn’t too into using protection, so I started to look into getting on PrEP… [When the nurse suggested it], I thought it might be a good idea. (P8)*

*Because of my sexual history and… the fact that I had a pretty active sex life, [the nurse] said PrEP was an option and asked me if I’d considered [using it]. (P4)*

Within these statements, it seems a momentary change in perspective at the point of being asked about PrEP opened an opportunity for participants to “consider PrEP” to increase their personal safety and reduce their risk of HIV. Regardless of their beliefs about their overall level of risk, being offered PrEP by nurses when they presented for sexual health visits (e.g., routine STI screening) led participants who were previously unsure about PrEP to consider this intervention.

#### Personal factors

Some participants described how their motivations to start PrEP related to more personal factors that led them to be concerned for their health. Several expressed “anxiety” and “worry” over the potential transmission of HIV from a sexual contact, which led some, like participant 11, to think “*PrEP might be something [they] would benefit from*”. Similar points were raised by participants who had previously used PEP and how their experience with an HIV “scare” led them to reconsider PrEP use. The following statements capture these sentiments:

*I had unprotected sex with someone I didn’t know very well. It came up in our encounter that this wasn’t the first time they’d had unprotected sex… Knowing about PEP, I further looked into PrEP and went to the Sexual Health Clinic. (P11)*

*There was a time where I was exposed unknowingly [to HIV], but I only found out months afterward unfortunately. (P7)*

*[It was] one of my rare occasions, receiving penetration and we did not use a condom. That got me very anxious. (P13)*

*I felt scared cause it only takes one time for anyone to get sick… After the whole drama of PEP and having to [assess the level of risk] of getting HIV… No, just give me the PrEP and I know how to deal with it. (P9)*

Evident in these quotes is that the experience of accessing PEP forced participants to re-evaluate what their level of risk might be. For these individuals, a “rare occasion” of condomless receptive anal sex or “unknowing exposure” to HIV led to a change in thinking about the effectiveness of their current risk reduction strategies and by extension, their stance on PrEP.

Building on this, other participants commented on how their motivation for PrEP came after a moment of introspection about the potential level of risk inherent in their sexual practices. For these individuals, the decision to use PrEP came after a period of reflection about their sexual behaviours and the risk these might pose to their health. On this point, participants stated:

*One of the main reasons I started to take PrEP… was because I had started a relationship… and it really freaked me out, the idea of passing him an infection. Like, it really bothered me. (P1)*

*I was struggling with drug and alcohol issues. So basically, anytime I would consume anything, I wouldn’t care about protection, so I would just not use it. At one point, I was even having impulse control issues with sex… so it was a combination of factors that made me think PrEP was something I needed to do for my health. (P6)*

Within this subset of participants, HIV-related stress stemming from the possible risk associated with their sexual practices became a personal motivation for PrEP. For these individuals, PrEP was a useful strategy to reduce the unknown variables in their current risk practices.

Finally, participants described other potential sources of HIV transmission that caused them to reconsider their HIV prevention strategies. These concerns related to the unknown risk behaviours of their sexual partners with respect to their STI/HIV status and sexual practices (e.g., condomless sex, drug use, etc.), which became a personal motivation for participants to add PrEP to their existing risk reduction plan. This point was noted in the following statements:

**What would make you anxious about sex? (I)**

*Well, specifically related to PrEP, would be HIV and other STIs. It’s still something I have some anxiety about. (P8)*

*It’s good that [PrEP] is protecting you from one thing, but the fact that you don’t even know if you have syphilis and still want to have unprotected sex… That’s kind of stupid. (P9)*

*In terms of STIs and HIV, I have no way of knowing ‘who’s who in the zoo’. (P2)*

For these participants, risk was not solely limited to the possibility of HIV transmission. Concerns about their sexual health extended to beliefs about the importance of consistent condom use to prevent HIV and STIs. Since secondary STIs can increase the likelihood of HIV acquisition (particularly among gbMSM), it is notable that participants remarked on the challenge of being able to assess, not only their partners’ level of HIV risk, but STI risk as well. In this way, PrEP (for HIV prevention) and condom use (for STI and HIV prevention) became a personal motivating factor for the use of PrEP as part of participants’ overall risk reduction strategy.

In summary, this theme explored the individual motivations for PrEP among PrEP-RN patients. Despite being offered PrEP by nurses, many participants expressed ambiguity about its use until a change in life circumstances (e.g., new sexual partner, change in risk practices), major HIV “scare” (resulting in them accessing PEP) or personal concern related to HIV acquisition (stemming from perceptions about partners risk practices or STI status) caused them to reconsider the potential benefits of PrEP. Implicit within these findings is that motivations for PrEP may not always relate to internal beliefs about risk. For participants in this study, motivation seemed to be affected by the timing of being offered PrEP by nurses and concerns about the threat of risk stemming, primarily, from the risk behaviours of others.

### Theme 2: beliefs about the benefits of PrEP

The second theme focuses on participants’ beliefs about the benefits of PrEP, beyond its pharmacologic indications. Indeed, informational campaigns and resources about PrEP typically advertise its benefits in terms of its ability to reduce the likelihood of HIV acquisition. Considering this, PrEP is often targeted at persons with inconsistent safe sex practices (e.g., multiple sex partners, condomless sex, partners living with HIV). However, despite awareness of the scientific data surrounding the efficacy of PrEP, participants in the PrEP-RN program described its benefits in different terms. These sentiments were divided into the sub-themes of: (1) circumventing mistrust, (2) taking control of sexual health, and (3) liberating fears surrounding HIV.

#### Circumventing mistrust

In terms of trusting partners, several participants spoke about the specific qualities they looked for in their sexual partners. These characteristics helped determine the “trustworthiness” of their partners and by extension, the level of risk these individuals might pose to their health. For many participants, these were traits they felt were evident during conversations with partners about their respective STI/HIV statuses and were captured in the following statements:

*Telling the truth and being integral is part of my core values. It makes me take the time to ask those questions before being willing to trust or engage in sex. (P13)*

*If the way they will portray themselves towards me is genuine, I know they aren’t lying to me. Somehow, in the back of my head, I know they’re being honest. (P9)*

*There is behaviour that is evident. It relies on perception of respect and of mutual trust and an aspect of perceived truthfulness and trustworthiness. You know, do things that people say bare out in my interactions with them. (P10)*

While discussions about STI/HIV history were useful for some participants to help determine the “trustworthiness” of potential sexual partners, for other participants, trust was more difficult to measure than in a blanketed conversation about one’s sexual health. Challenges around assessing whether these partners were in fact being “truthful” or “genuine” about being HIV-negative led to persistent worries, which were captured by participants as follows:

*As much as I do ask people what their sexual health habits are, people can lie. (P5)*

*Both with partners in a formal relationship or in a casual hookup, it’s hard to trust that people are disclosing the facts, or in turn, if they actually know [what their HIV status is]. (P4)*

*People who are HIV-positive are a lot safer to have sex with than someone who claims they’re HIV-negative and has no idea. (P2)*

*It got to the point where even if I met somebody who said they were negative, but weren’t on PrEP, I couldn’t trust them. I couldn’t believe them. (P6)*

For these individuals, establishing whether sexual partners were “disclosing the facts” about their HIV status became a near impossibility due to persistent unease these partners might be “lying”. Concerns about potential omissions of truth were not, however, strictly limited to partners deliberately claiming to be HIV-negative when they were actually HIV-positive. In these statements, participants primary fear was that partners who had not completed HIV testing or had a confirmatory negative result, would claim to be HIV-negative based on lack of symptoms or beliefs about their own level of risk, which participants felt was difficult to mitigate.

#### Taking control of sexual health

To help reconcile their potential HIV risk (rather than relying exclusively on sexual partners’ self-reported HIV status and risk practices), several participants turned to PrEP as a means of managing their health. Based on the statements made by participants, PrEP assisted these individuals in reducing their anxiety about HIV, increasing confidence about their sexual health status, and protecting their sexual partners from a potential HIV transmission. For participants, PrEP was more than a chemoprophylactic intervention; it seemed to have physiologic and psychologic benefits that helped control their perceived level of HIV-related risk. Indeed, many participants spoke of the anxiety relieving effects of PrEP use, which were exemplified in the following statements:

*There’s lots of people who have been surprised [by an HIV diagnosis]. So, it’s just something that kind of hangs over you… PrEP is a terrific sexual anxiolytic. (P12)*

*I was already very anxious*. I had a lot of uncertainty, so [PrEP] helped calm me down, to know that I was doing whatever I could to control that situation. (P14)

*A lot of things make me paranoid about diseases and stuff, so anything to relieve any of that stress about meeting guys makes it so much better for me mentally. (P3)*

*It’s nice to have a safety net… where I can explore my relationship and not have to feel anxiety every time I want to have sex. (P7)*

Implicit in these statements is the ability of PrEP to “relieve stress” about HIV transmission. For many of these participants, persistent worries about HIV, STIs, and risk caused them to be in a seemingly constant state of “anxiety” during sex, which in some cases, impacted their mental wellbeing. In this way, PrEP acted as more than an HIV prevention tool, it was also an anxiolytic because it helped participants “control the uncertainty” they felt about engaging in casual sex.

Other participants expressed slightly different views about how PrEP made them feel. In this case, ‘taking control’ related to the reassurance PrEP provided about their sexual health (e.g., STI/HIV status), which was bolstered by its proven ability to reduce the likelihood of HIV acquisition. On this point, participants stated:

*[PrEP gave me] a bit more control and power in managing the level of risk while I’m having sex. (P4)*

*It takes [stress] out of having to trust, sometimes a stranger, and puts the control in my hands… that’s power in my control. (P5)*

*If we were to find ourselves in a situation where we were interested in doing something with others, there would at least be that level of reassurance… that the HIV aspect of it would be greatly reduced. (P10)*

For these individuals, a primary benefit of PrEP was that it provided them with a level of comfort and “control” in terms of “managing risk” and “reducing HIV”. This led participants to feel more empowered in their decisions around engaging in sexual activity with other men or about pursuing relationships with sexual partners who might not be aware of their own HIV status.

The “power” elicited through “managing risk” by using PrEP also extended to feelings about the protective benefits of PrEP to safeguard the health of sexual partners. Specifically, participants described how the “reassurance” PrEP gave them about their own HIV (negative) status led them to feel more confident they would not inadvertently transmit HIV to their partners. These sentiments were captured in the following statements:

*The fact that I’m on PrEP makes me reassured that I’m not going to transmit HIV and at the same time it tells the other person that I’m taking care of myself to protect me and protect them as well. (P9)*

*[PrEP] allowed me to take control of my sexual health and have conversations about sexual health… Here’s what I’m doing to ensure I’m safe and keeping you safe. (P2)*

Within these excerpts, “safety” was not limited to participants personal health and wellbeing. Several remarked on the ability of PrEP to “protect [themselves] and others”, which made them feel more self-assured engaging in sexual activities with new or regular sex partners. Thus, while many participants spoke of their beliefs about the benefits of PrEP to manage their own level of risk, the “safety” of their sexual partners was noted to be of equal importance.

#### Liberating fears surrounding HIV

Building on the notion of increased comfort to engage in sexual practices, beliefs about the benefits of PrEP also extended to feelings of liberation this medication provided. For some participants, PrEP helped relieve some of the tension and anxieties they sometimes experienced during “hookups” with casual sex partners. For others, liberation related to the feeling of freedom PrEP gave them to engage in the types of sexual practices they were interested in but had previously avoided due to internal constraints over a potential HIV exposure.

Many participants also spoke of the liberation elicited by PrEP following sexual contact with a new partner. For these individuals, PrEP did more than quell their concerns about HIV, it also helped increase their comfort about engaging in casual sex. On this point, participants stated:

*[PrEP gave me] a level of comfort… If I would have a hookup, my brain would always wonder, ‘What if the condom broke?’… Ever since PrEP, it was like no, done. (P14)*

*It lifted that dark cloud over my shoulders, and I found it very liberating… It [gave] me the reassurance of knowing I didn’t have to worry about HIV. (P2)*

*I’m definitely more comfortable having sex… whereas before, I had some hesitations… It’s an empowering thing and responsible choice for some. (P4)*

Among participants in this review, PrEP helped to “lift the dark cloud” (i.e., anxiety related to HIV acquisition) that had once restrained their sexual activity. In this case, PrEP made participants feel “more comfortable” during sex, particularly when these encounters did not follow their typical risk reduction strategies (e.g., condom use, discussions with partners in advance, etc.).

In addition, some participants spoke of the benefits of PrEP for giving them increased sexual liberties to experiment with new sexual practices during their sexual encounters. In brief, PrEP “*made sex more fun*” (P12), which many participants felt was both exciting and unfamiliar to them. On the point of their new-found sexual liberation, participants stated:

*Before, I would have been pretty insistent about condom use. Now, it’s given me the option not to. (P5)*

*It gives you freedom and creates opportunities for you to explore your sexuality. (P2)*

*[My feelings about condom use] changed over time to feeling more comfortable having unprotected sex with partners. (P4)*

*It certainly lowered by guard if I saw [a sexual partner] was on PrEP. I felt more comfortable to engage in bareback. (P6)*

While many participants noted their use of condoms did not change dramatically because of PrEP, this intervention gave participants the “freedom and opportunity” to explore personal boundaries and experiment sexually with new partners. In this way, PrEP helped to liberate participants by “lower[ing] their guard” and allowing them to be “more comfortable” with the notion of risk.

In this theme, several participants noted the personal importance of having discussions about their STI/HIV status with partners prior to engaging in sex. However, many raised concerns about their ability to know if these partners were sufficiently “genuine” or “trustworthy” to have sex with, which left them with feelings of mistrust. For these individuals, PrEP provided reassurance about their sexual health and sexual practices, which, in turn, gave them “power” and “control” over their perceived risk of HIV acquisition or potential transmission to partners. In this way, PrEP also “liberated” participants’ feelings of anxiety around the possibility of a sexual exposure to HIV and gave them the “freedom” to engage in the types of sexual practices (e.g., bareback sex), they found pleasurable, but had once avoided due to concerns about HIV.

## Discussion

This paper presents on the findings from qualitative interviews completed with participants in a nurse-led PrEP program, known as PrEP-RN. Among our participants, the decision to initiate PrEP was influenced both by external life factors (e.g., access to insurance or the increasing popularity of PrEP) and personal perceptions of risk stemming from the experience of using PEP and health concerns centered around HIV and STIs (Theme 1). Regular and consistent PrEP use helped mitigate feelings that sexual partners might be untruthful or unaware of their HIV status (Theme 2). At the same time, participants described their beliefs that PrEP enabled them to take control over their sexual health by feeling more prepared during sexual encounters, which reduced anxieties or misgivings they felt about engaging in the sexual practices they enjoyed (Theme 2). These findings raise some interesting points for healthcare providers and allied workers providing sexual health and HIV prevention services to persons from priority groups.

The first point of interest from these findings relates to the difference in perceptions of HIV risk between patients and healthcare providers. Specifically, our participants were offered referrals for PrEP based on nurses’ identification of practices that could put these individuals at increased risk of HIV acquisition. However, many participants seemed incognizant that they might be at risk (based on scientific estimates) until an unforeseen scenario arose (e.g., PEP use, condomless sex, anonymous partner), which forced self-reflection. This finding aligns with literature surrounding perceptions of risk among gbMSM, which shows that while persons in this group consider themselves at low to moderate risk for HIV, healthcare providers often place these individuals in higher risk brackets [27–29]. Moreover, individual perceptions of risk can vary based on the type of sexual practices an individual chooses to engage in, with most preoccupations of HIV-related risk centering around condomless receptive anal sex [30, 31]. For our gbMSM participants then, it appears instead that risk calculations were stratified based on an intrinsic threshold of HIV risk, which established personal boundaries for sexual encounters. Beliefs about risk and potential for HIV exposure were subsequently manifested when sexual acts crossed the upper limit of these risk thresholds. In this regard, patients’ perceptions of risk are often highly subjective and context dependent—and thus not necessarily aligned with healthcare provider’s views.

However, a caveat exists within healthcare provider’s assessments of risk, which is that many risk tools to assess PrEP candidacy, such as the HIRI-MSM, use validated metrics which can inflate calculations of risk among gbMSM based solely on their sexual orientation and a single episode of condomless receptive anal sex [32]^.^ While this act can pose a higher risk for HIV, such a score suggests that any individual who engages in such behaviour is automatically ‘risky’ and in need of intervention (i.e., PrEP). In this way, subjective risk scoring systems can put healthcare providers’ views ahead of patients’ views, which can lead to hierarchies of risk knowledge. In other words, healthcare providers, as professionals with expert knowledge and training in health, gain final determination of who ‘should’ or ‘can’ obtain PrEP based on their subjective calculations of risk. Though both patients and healthcare providers engage in individual risk assessments based on preconceived ideas about HIV acquisition (where for patients, risk is calculated based on personal thresholds and for healthcare providers, risk is calculated based on scientific estimates), the latter is seen as a more accurate assessment of PrEP candidacy.

The discussions above are explained in previous work by Lupton [32] regarding the limitations of public health education. Specifically, hierarchies in risk knowledge are established, whereby experts, such as nurses, construct perceptions of risk and engage in health mediating interventions (e.g., education, condom campaigns, PrEP promotion, etc.) to correct behaviour that deviates from the imperative of health [32]. In this so-called ‘expert’ construction of risk, healthcare providers impose their views of health (based on clinical knowledge of risk) onto patients to modify aberrant health behaviours [32]. Hierarchies of risk knowledge are perpetuated during healthcare visits by establishing an ‘ideal’ model of behaviour (generally centered around outright avoidance of risk) [22] and instructing patients to take corrective actions to ‘be healthy’. While clinically precise and scientifically robust, healthcare estimates of risk fail to recognize the intricacies of personal subjectivity in risk-taking [32, 33], such as the thrill of meeting an anonymous sex partner for the first time or the pleasure elicited from condomless (bareback) sex. The current use of risk scores to determine PrEP eligibility are an ongoing example of comments raised by Lupton [32] over 25 years earlier—highlighting that healthcare assessments of, and patient education about, risk have remained relatively unchanged. Thus, in order to advance PrEP uptake among priority groups, a change in approach might be warranted.

The second point of interest from these findings builds on the foregoing discussions about individual perceptions of risk. Given the varied ways in which patients construct personal risk thresholds, another strategy to increase PrEP uptake would be to re-structure the way health and allied providers promote PrEP to their patients. Indeed, current messages in promotional campaigns about PrEP tend to narrow in on its protective abilities and its potential to reduce HIV acquisition and maintain one’s health [34–36]. Despite these pointed promotional messages designed to increase PrEP uptake, it is notable that global estimates of PrEP show 42% of gbMSM who are offered PrEP decline this intervention [37]. This figure aligns with data from the PrEP-RN study which found 52% of participants with objective risk factors for HIV who were offered PrEP opted not to start [20]. Of pressing concern, however, is that PrEP uptake among other priority groups, such as Indigenous communities, persons from HIV endemic countries, trans men and women, and people who use drugs are much lower than those of gbMSM, yielding an estimated 3–23% acceptance rate [18, 19]. This minimal figure signals that more work needs to be done to encourage PrEP among individuals outside of white, gay men—who might be within the target demographic for PrEP but are not the only group who could benefit from this intervention.

Given that current efforts to increase PrEP uptake results in an acceptance rate of less than 50% among persons with elevated group incidences of HIV, it might be prudent for healthcare providers and allied HIV prevention workers to incorporate patients’ perspectives into their current PrEP promotion strategies. Building on the sentiments conveyed by participants in this study, one such strategy could be to discuss the personal benefits provided by PrEP, rather than to strictly focus on the population benefits of reduced HIV infection. For example, discussions could include the reassurance provided by more frequent STI/HIV testing (with screening done every three months as part of PrEP care) and increased personal confidence and knowledge related to sexual health. In addition, providers should consider discussing the benefits PrEP can provide for patient’s sex lives. For example, PrEP can increase sexual liberties, enhance pleasure (due to reduced anxiety and heightened sensation) and provide greater comfort to experiment with a variety of sexual practices. While not markedly different than some of the points taken from current PrEP information websites, a slight change in language and syntax could produce messages that might be less stigmatizing and as a result, may lead to better uptake of PrEP among persons from priority groups. Simply put, patients’ motivations for PrEP are different from what healthcare providers think these are, signaling that, to increase PrEP uptake, providers need to consider changing the way they communicate information about PrEP.

While incorporating patients’ subjectivity into PrEP counselling might be a useful measure to increase PrEP uptake, healthcare providers must also recognize that even when appropriate counselling is provided or subsidized medications are offered (as was the case for PrEP-RN patients), not all patients will choose to initiate PrEP. Indeed, among participants in this study, many did not see the perceived benefits of PrEP until after they had started taking it, which could lead patients to reflexively dismiss this option during healthcare visits. For this reason, when counselling patients about PrEP, healthcare providers should ensure those who decline a PrEP referral also receive information about the indications for, and availability of, PEP [32]. In addition, any patient who is initiated on PEP should receive counselling about the option of PEP2PrEP [38] (i.e., transitioning to PrEP immediately after the completion of PEP).

### Limitations

This study has some limitations. First, it occurred in a single Canadian city and involved a sample consisting of mostly white gay men. These results could have differed had the study sample included greater ethnic and gender diversity. Second, at the time interviews were conducted, half of these participants had stopped using PrEP. While several patients noted this decision was the result of reduced sexual activity due to the COVID-19 pandemic (and coinciding stay-at-home orders), motivations for PrEP were not necessarily an unremitting factor for all participants. Third, nearly half (n = 6/14) of our participants had previously used PEP, meaning that these individuals may have had a lower level of risk tolerance from seeking emergency HIV prophylaxis, compared to participants who were referred for STI diagnoses or based on nurses’ clinical judgement. Lastly, that PrEP was subsidized for participants could have also affected uptake, which was noted in some participants statements surrounding motivations for PrEP related to obtaining this medication at low to no cost.

## Conclusion

The findings from qualitative interviews completed with participants in the nurse-led HIV prevention program, PrEP-RN, raised some important points of consideration for health and allied providers. Specifically, personal motivations for PrEP and beliefs about the benefits of PrEP differed from ‘expert’ or scientific assessments of HIV-risk that dominate mainstream discourses. Further research is required about how to effectively engage in discussions about PrEP with patients in a way that resonates with their personal construction, or beliefs, about HIV risk. From a healthcare perspective, these sentiments could be used to improve risk counselling messages with patients during health visits or added to information pamphlets given to patients who are considering PrEP. From a health promotion perspective, patient perceptions about the benefits of PrEP could be added to websites, promotional materials, and public education campaigns around sexual health. A change in strategy could result in increased awareness and introspection around the utility of PrEP, particularly for individuals who are unaware or ambivalent about this option.

Finally, it is important for providers to consider systemic inequities to accessing HIV prevention services among priority groups, where PrEP uptake is markedly higher among white, gay men compared persons of African, Caribbean, or Black descent, Indigenous communities, trans persons, and people who use drugs. The simple fact remains that in Canada, HIV rates continue unabated among these priority groups. Widespread PrEP implementation, if rolled out well, should attempt to incorporate findings such as these—which include patient subjectivities in assessments of risk and personal motivations for PrEP—and might help reduce these rates.

## Abbreviations

PrEP: Pre-exposure prophylaxis
PEP: Post exposure prophylaxis
gbMSM: Gay, bisexual, and other men who have sex with men
HIV: Human immunodeficiency virus
STI: Sexually transmitted infection(s)
